# Influence of SLM-, SLS-, and DMLS-Manufactured Titanium Meshes on Bone Gain Parameters and Complications: A Systematic Review

**DOI:** 10.3390/dj13090387

**Published:** 2025-08-26

**Authors:** Viktor Savov, Stefan Peev, Ralitsa Yotsova, Varvara-Velika Rogova

**Affiliations:** 1 Department of Periodontology and Dental Implantology, Faculty of Dental Medicine, Medical University of Varna, 9002 Varna, Bulgaria; stefan.peev@mu-varna.bg; 2 Department of Oral Surgery, Faculty of Dental Medicine, Medical University of Varna, 9002 Varna, Bulgaria; ralitsa.yotsova@mu-varna.bg (R.Y.); varvara.rogova@mu-varna.bg (V.-V.R.)

**Keywords:** customized titanium mesh, individualized titanium mesh, augmentation, bone grafting, alveolar ridge

## Abstract

**Background/Objectives**: Guided bone regeneration one of the most widely used techniques, relies on combining bone graft material with barrier membranes or meshes. The choice of the mesh material depends on the specific clinical situation. Among the available options, titanium membranes are recognized as one of the most effective in dental implantology. The latter can be categorized into two groups: commercial and individualized. Advancements in additive manufacturing make customized titanium meshes an attractive option for bone regeneration. Customized titanium meshes can be manufactured using three main methods: selective laser sintering (SLS), selective laser melting (SLM), and direct metal laser sintering (DMLS). This review aims to provide information about the differences between the production process and the clinical outcomes. **Methods**: This systematic review was conducted by the Preferred Reporting Items for Systematic Reviews and Meta-Analysis for Scoping Reviews (PRISMA-ScR). Relevant articles were sought out in the Web of Science, PubMed, and Scopus databases. **Results**: A total of ten articles were included and thoroughly reviewed. The type of bone graft used, the manufacturing technique, the amount of bone gain, the healing time, and the intraoperative and postoperative complications are discussed. **Conclusions**: All the relevant studies demonstrated good and predictable results using augmentation with individualized titanium meshes manufactured via SLS, SLM, or DMLS methods.

## 1. Introduction

The rehabilitation of partially or fully edentulous patients with dental implants has become a cornerstone in modern dentistry. Implant treatment is rapidly becoming the most sought-after method for restoring the functionality of lost structures [1]. However, there are many obstacles in the way of placing a prosthetic-guided implant. One of the biggest challenges is insufficient alveolar bone volume, and among the most frequent causes thereof were trauma, periodontal diseases, and long-term edentulism [2]. The adequate volume of bone is the basis for a good prognosis and long-term, successful functional and aesthetic results in prosthetic-guided implantology [3,4].

Various augmentation techniques are applied in modern implant dentistry. Guided bone regeneration (GBR) is a method in which augmentation of all or part of the alveolar crest is performed using barrier membranes and bone materials [5,6,7,8,9]. However, not every clinical case is suitable for the GBR technique with artificial bone and resorbable or non-resorbable membrane. The non-resorbable membranes are the expanded and dense types of titanium-reinforced polytetrafluoroethylene membranes and titanium meshes (commercial or customized) [10]. Titanium meshes possess excellent mechanical properties and are used in large bone defects where more stable and resistant membranes with enough malleability and hardness are needed to reconstruct the bone volume [10,11]. Their rigidity can maintain the required space for an extended period and prevent the augmented area from collapsing, while their stability helps hold the graft material in place [12,13].

Titanium meshes used for bone augmentation in dental implantology can be divided into two groups: commercial and customized/individualized. Commercial membranes are available in the form of plates that can be used in different areas of the edentulous ridge. Before use, however, they need to be cut to perfectly fit the augmented area. This time-consuming and skills-demanding trimming process carries various hazards. The newly formed edges of the titanium membranes can cause damage to surrounding soft tissues, leading to mesh exposure and, consequently, an increased risk of infection and a possible failure of the GBR procedure [14,15]. The disadvantages of the commercial membranes led to the search for a more patient-specific approach. With the development of computer-aided design and computer-aided manufacturing (CAD-CAM), the accuracy of pre-operative planning has significantly increased. 

Additive manufacturing (AM) is the final step that finishes the digital workflow—it connects disease diagnosis, treatment planning, and production, thus creating a fully digital working protocol [16,17]. AM is a technique for producing physical objects with exact structure and shape from CAD data [18]. The method includes different layer-by-layer production-based technologies that generate a physical model directly from the CAD system. 

Selective laser sintering (SLS) is a type of PBF that uses one or more laser beams to selectively fuse powder materials and produce an object in a closed chamber. This process is used in the production of a wide range of materials, including polymers, metals, ceramics, and their composites, making it suitable for individualized applications [19].

Selective laser melting (SLM) is another type of powder bed fusion technique that likewise works layer-by-layer, yet only with metal materials. The heat source in SLM is again a laser beam that melts the surface metal powder and merges a layer. SLM overcomes many limitations of the conventional processes. First and foremost, it can produce high-detailed structures with complex geometries [20,21], and precisely for this reason, it is being used more and more widely [22].

Direct metal laser sintering (DMLS) is a similar process to SLM that only works with metal alloys and powder-combined metals. The method is adapted from the SLS and uses a laser beam to condense metal powder without binders [23]. When the process is finished, the unused powder is kept until a new production cycle (waste storage phase) [19].

The present review discusses the parameters of the augmented bone and complication rate of the guided bone regeneration procedure using customized titanium mesh manufactured with SLS, SLM, or DMLS methods. It aims to analyze the effectiveness of this augmentation method by taking into consideration the bone volume gained and complications that can occur during the surgery or the healing phase.

## 2. Materials and Methods

The present systematic review was guided by the PIO framework and addressed the following components:

**P (Patient/Population/Participants)**—patients with bone deficiency.

**I (Intervention/Exposure)**—augmentation with customised titanium meshes produced with the SLS, SLM, or DMLS technique.

**O (Outcome)**—reported complications and parameters of the augmented bone volume.

**Review Question:** what are the reported complications and parameters (O) when doing augmentation with customized titanium meshes produced with the SLS, SLM, or DMLS technique (I) in patients with bone deficiency?

### 2.1. Eligibility Criteria

The inclusion criteria were as follows: clinical studies discussing individualized meshes produced by the SLS, SLM, or DMLS method and including the following information: area of augmentation, gained bone volume, and complication rate.

The exclusion criteria were as follows: review articles, books, book chapters, abstracts, and clinical studies not discussing individualized meshes produced by the SLS, SLM, or DMLS method and not including the following information: area of augmentation, gained bone volume, and complication rate.

### 2.2. Information Sources

This systematic review was conducted in accordance with the Preferred Reporting Items for Systematic Reviews and Meta-Analysis for Scoping Reviews (PRISMA-ScR) [24,25]. It was registered in the Open Science Framework (OSF) and can be found at https://archive.org/details/osf-registrations-5f82e-v1 accessed on 12 June 2025.

A thorough search for relevant research articles was carried out electronically on 14 June 2025, in the Web of Science, PubMed, and Scopus databases.

### 2.3. Search Strategy

The research approach involved conducting an advanced search in the chosen databases by employing various combinations of keywords. Only full-sized articles written in English were included to gather the latest evidence, highlighting recent developments in the subject area.

The keywords used in the Web of Science database were **(((((TS = (customized titanium mesh)) OR TS = (individualized titanium mesh)) AND TS = (augmentation)) OR TS = (bone grafting)) OR TS = (bone substitute)) AND TS = (alveolar ridge)** and **Article** (Document Types) and **English** (Languages).

The keywords used in the PubMed database were **((((((titanium mesh) OR (individualized titanium mesh)) OR (customized titanium mesh)) AND (augmentation)) OR (bone graft)) OR (bone substitute)) AND (alveolar ridge)** Filters: **Clinical Trial, Randomized Controlled Trial, English.**

Expanded search: ((((((“titanium”[Supplementary Concept] OR “titanium”[All Fields] OR “titanium”[MeSH Terms] OR “titanium s”[All Fields] OR “titaniums”[All Fields]) AND (“medical subject headings”[MeSH Terms] OR (“medical”[All Fields] AND “subject”[All Fields] AND “headings”[All Fields]) OR “medical subject headings”[All Fields] OR “mesh”[All Fields])) OR ((“individual s”[All Fields] OR “individualisation”[All Fields] OR “individualise”[All Fields] OR “individualised”[All Fields] OR “individualising”[All Fields] OR “individualism”[All Fields] OR “individualisms”[All Fields] OR “individualities”[All Fields] OR “individuality”[MeSH Terms] OR “individuality”[All Fields] OR “individualization”[All Fields] OR “individualize”[All Fields] OR “individualized”[All Fields] OR “individualizes”[All Fields] OR “individualizing”[All Fields] OR “individually”[All Fields] OR “individuals”[All Fields] OR “individuate”[All Fields] OR “individuated”[All Fields] OR “individuates”[All Fields] OR “individuating”[All Fields] OR “individuation”[MeSH Terms] OR “individuation”[All Fields] OR “individuations”[All Fields] OR “persons”[MeSH Terms] OR “persons”[All Fields] OR “individual”[All Fields]) AND (“titanium”[Supplementary Concept] OR “titanium”[All Fields] OR “titanium”[MeSH Terms] OR “titanium s”[All Fields] OR “titaniums”[All Fields]) AND (“medical subject headings”[MeSH Terms] OR (“medical”[All Fields] AND “subject”[All Fields] AND “headings”[All Fields]) OR “medical subject headings”[All Fields] OR “mesh”[All Fields])) OR ((“culture”[MeSH Terms] OR “culture”[All Fields] OR “custom”[All Fields] OR “customs”[All Fields] OR “customer”[All Fields] OR “customer s”[All Fields] OR “customers”[All Fields] OR “customization”[All Fields] OR “customizations”[All Fields] OR “customize”[All Fields] OR “customized”[All Fields] OR “customizes”[All Fields] OR “customizing”[All Fields]) AND (“titanium”[Supplementary Concept] OR “titanium”[All Fields] OR “titanium”[MeSH Terms] OR “titanium s”[All Fields] OR “titaniums”[All Fields]) AND (“medical subject headings”[MeSH Terms] OR (“medical”[All Fields] AND “subject”[All Fields] AND “headings”[All Fields]) OR “medical subject headings”[All Fields] OR “mesh”[All Fields]))) AND (“augment”[All Fields] OR “augmentation”[All Fields] OR “augmentations”[All Fields] OR “augmented”[All Fields] OR “augmenting”[All Fields] OR “augments”[All Fields])) OR (“bone transplantation”[MeSH Terms] OR (“bone”[All Fields] AND “transplantation”[All Fields]) OR “bone transplantation”[All Fields] OR (“bone”[All Fields] AND “graft”[All Fields]) OR “bone graft”[All Fields]) OR (“bone substitutes”[Supplementary Concept] OR “bone substitutes”[All Fields] OR “bone substitute”[All Fields] OR “bone substitutes”[MeSH Terms] OR (“bone”[All Fields] AND “substitutes”[All Fields]) OR (“bone”[All Fields] AND “substitute”[All Fields]))) AND (“alveolar process”[MeSH Terms] OR (“alveolar”[All Fields] AND “process”[All Fields]) OR “alveolar process”[All Fields] OR (“alveolar”[All Fields] AND “ridge”[All Fields]) OR “alveolar ridge”[All Fields])) AND ((clinicaltrial[Filter] OR randomizedcontrolledtrial[Filter]) AND (english[Filter])).

The keywords used in the Scopus database were titanium OR titan OR ti AND mesh OR framework OR scaffold AND bone OR osseous OR osseointegration AND augmentation OR grafting OR repair OR regeneration AND customized OR individualized AND (LIMIT-TO (DOCTYPE, “ar”)) AND (LIMIT-TO (LANGUAGE, “English”)).

### 2.4. Study Selection, Data Collection, and Data Items

Details such as titles, abstracts, author names, and publication years of the identified studies were compiled into an MS Excel spreadsheet, and the duplicates were removed afterwards. Two independent reviewers (V.S. and R.Y.) screened titles and abstracts for eligibility before assessing full-text articles against the predefined inclusion and exclusion criteria. Extracted data included authors, publication year, study models, sample size, bone volume gained, bone graft material, complication rate, healing time, area of augmentation, and method of mesh production. Disagreements between researchers were resolved through discussion, consensus, or, if needed, arbitration by a third reviewer (S.P.).

### 2.5. Risk of Bias Assessment

For the purpose of this review, a quality assessment was conducted using these quality assessment tools: Cochrane RoB2 for RCT articles [26], JBI Critical Appraisal Tool for Case Reports [27], and JBI Critical appraisal Tool for Case series [28].

## 3. Results

**Figure 1:**
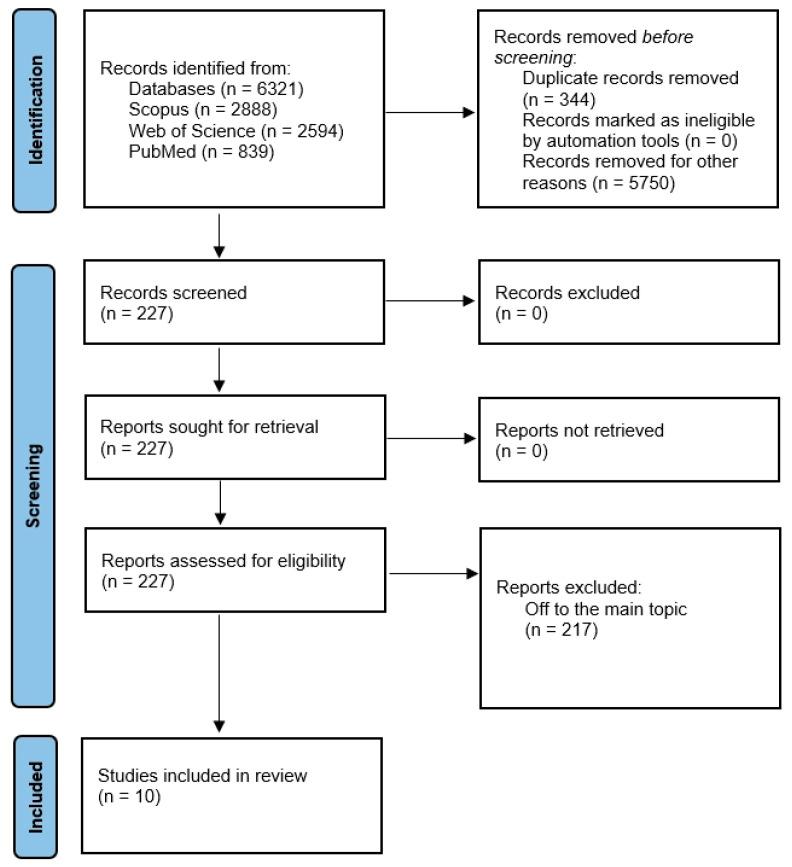
PRISMA flow diagram of the research.

The article selection process is graphically presented in Figure 1. In the preliminary search, 6321 studies were found to be potentially relevant. After the exclusion of 344 duplicates, 5977 articles remained. Those that did not meet the eligibility criteria were excluded, and 10 articles were considered to be included in the review. A total of ten original articles were included in the present review.

**Figure 2:**
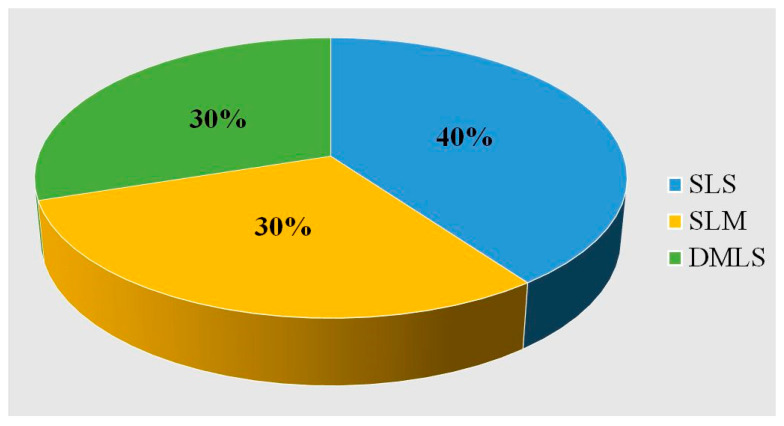
Distribution of production methods (selective laser sintering, SLS; selective laser melting, SLM; or direct metal laser sintering, DMLS).

In the selected articles, the most commonly applied technique of customized titanium mesh production (in 4 out of 10 studies) is that of SLS (Figure 2).

The data considering bone gain and type of bone graft are summarized and presented in Table 1.

**Figure 3:**
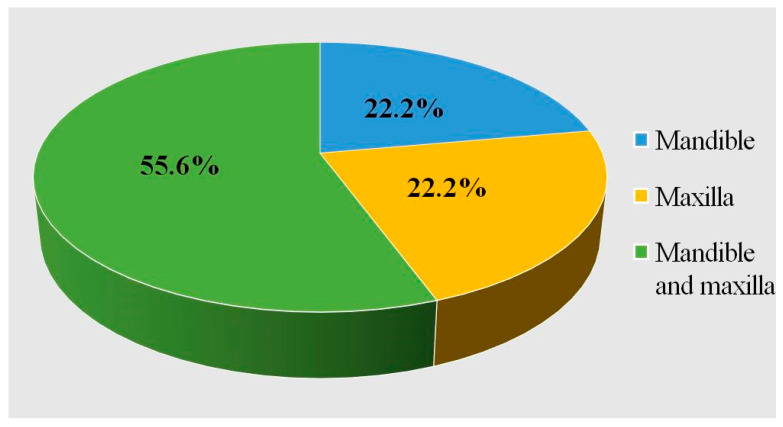
Augmented area distribution (mandible, maxilla, or mandible and maxilla).

Considering the area of augmentation, most studies include augmentation of both maxilla and mandible (Figure 3).

The information about the healing time and type, and the occurrence of complications is given in Table 2.

### Risk of Bias Assessment

**Figure 4:**
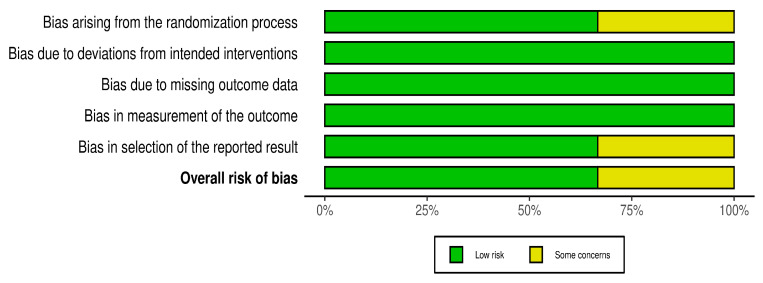
Risk of bias assessment for RCT.

With regard to RCT articles (Figure 4), the overall risk of bias was assessed as low. Only one study was reported, with some concerns due to bias arising from the randomization process and bias in the reported results.

With regard to case reports, the overall risk of bias was assessed as low (Table 3).

Traffic light charts for the risk of bias assessment of individual RCT studies can be found in the **Supplementary Materials**, as well as PRISMA checklist.

With regard to case series, the overall risk of bias was assessed as low (Table 4).

## 4. Discussion

### 4.1. Method of Mesh Production

#### 4.1.1. Selective Laser Sintering

Chiapasco et al. conducted a retrospective clinical study on 41 patients with 53 severely atrophic alveolar ridge sites requiring vertical and horizontal bone regeneration. Customized titanium meshes were produced via SLS, filled with a 1:1 mixture of autogenous bone particles and bovine bone mineral, and covered with collagen membranes. After an average healing time of 7.3 months (5 ÷ 12 months), the meshes were removed. A total of 106 implants, pure titanium or titanium alloys, were put in their place, and uncovered after a mean of 3.5 months (2 ÷ 5 months). The study reported mean vertical (VBG) and horizontal bone gain (HBG) of 4.78 ± 1.88 mm and 6.35 ± 2.10 mm, respectively, with a 100% implant survival rate after a mean follow-up of 10.6 months. Complications included varying degrees of mesh exposure in 11 of 53 sites (20.7%), with only one case considered a failure [32].

The 2021 cohort study by Dellavia et al. evaluated the histological quality of alveolar bone regenerated using customized SLS-produced titanium meshes in 20 partially edentulous patients with severe posterior mandibular atrophy. The guided bone regeneration procedure involved applying a mixture of autologous bone particles and deproteinized bovine bone matrix in equal quantities beneath the customized titanium membrane. Bone biopsies were collected for histomorphometry after mesh removal at the end of the 9-month healing period. According to the obtained results, the implant placement was successful in all patients (except in the three cases of membrane exposure), with the titanium mesh well osseointegrated. Histological analysis revealed that the regenerated tissue was highly mineralized and well-organized, and quantitatively, it comprised 35.88% new lamellar bone, 16.4% woven bone, 10.9% osteoid matrix, 14.1% residual graft material, and 22.7% medullary spaces [34].

A non-inferiority randomized clinical trial by Cucchi et al. involved 50 patients with vertical alveolar bone defects. A total of 48 individuals were randomly treated with either titanium-reinforced dense polytetrafluoroethylene mesh (control group), covered with a pericardium collagen membrane, or customized SLS-made titanium mesh, coated with a native collagen membrane. Grafting was conducted with a mixture of autogenous bone particles and deproteinized bovine bone matrix in a 1:1 ratio. After a healing period of 6 months, outcomes measured included surgical and healing complication rates, VBG, regenerated bone volume, and regeneration rates. The study found surgical complication rates of 4% after using Teflon mesh and 12% after using customized titanium membrane, with healing complication rates at 12.5% and 8.3%, respectively. In the control group, the regeneration rate was significantly higher (99.5%) compared to that in the test group (87.0%). However, the average VBG (5.79 ± 1.71 mm in the control group and 5.18 ± 1.61 mm in the test group) was statistically comparable. Similar values were also presented concerning implant osseointegration (~98%) and implant stability [37].

In a 2024 randomized clinical trial by Giragosyan, Chenchev, and Ivanova compared vertical and horizontal alveolar ridge augmentation outcomes using SLS-made titanium meshes versus titanium-reinforced dense polytetrafluoroethylene membranes (control) in 40 patients with deficient alveolar ridges. The study involved guided bone regeneration with a 1:1 mixture of xenograft and autogenous bone. A comparable bone height gain was achieved in both groups (3.65 ± 1.73 mm for titanium mesh vs. 4.24 ± 2.19 mm for Teflon mesh). Moreover, no significant differences were found for bone width gain (2.48 ± 1.03 mm in the test group vs. 2.60 ± 0.82 mm in the control group). Postoperative complication rates were also similar (33.3% and 38.9%, respectively) [38].

In the first two articles were presented and discussed the results of the augmentation of the alveolar bone using customized titanium meshes aided by the SLS method. It was stated that the effectiveness of the SLS method is undeniable, although complications related to mesh exposure were presented.

In the last two studies, results show that, compared to titanium-reinforced dense polytetrafluoroethylene membranes, titanium meshes presented a higher rate of intraoperative complications; however, the healing process appears to be more successful when using SLS.

#### 4.1.2. Selective Laser Melting

Another clinical trial of Cucchi et al. included a prospective randomized model with a sample size of 30 patients with partial edentulism of the maxilla or mandible, divided into two groups: one treated with customized SLM-designed mesh alone and the other with the mesh plus cross-linked collagen membrane. Grafting was performed using a 50:50 autogenous bone and xenograft material mixture. After a 6-month healing period, patients in the mesh plus membrane group showed fewer healing complications (13%) compared to those in the other group (33%), though this difference was not statistically significant. Regenerated bone volume averaged 843.13 mm^3^ when mesh plus membrane was used and 803.27 mm^3^ when mesh was applied alone, with comparable regeneration rates of 82.3% and 74.3%, respectively. VBG and implant survival rates were similarly comparable, with 71 implants placed and 68 of them stable at follow-up [33].

Gelețu et al. described the utilization of customized titanium mesh for complex alveolar bone defect regeneration. The case report concerned a 27-year-old female patient following a deficient odontectomy. The bone defect, measuring approximately 4.19 mm in length and 4.2 mm in width, was augmented using a titanium mesh fabricated via SLM. The graft consisted of particulate bone mixed with autogenous bone chips. After mesh fixation and a healing period of 6 months, a favorable bone width and height gain sufficient for implant placement was established. The procedure was not complicated by significant adverse events, and the mesh effectively maintained space for bone regeneration in this challenging aesthetic area [35].

Kurtiş et al. presented the successful vertical bone augmentation with a customized titanium mesh. A severe alveolar ridge defect in the posterior mandible of the included patient was caused by implant removal due to advanced periimplantitis. The study involved a customized titanium mesh fabricated via SLM covered with a collagen membrane. The bone graft consisted of a combination of autogenous bone, deproteinized bovine bone mineral, solid advanced platelet-rich fibrin, and injectable platelet-rich fibrin. After the set healing period (9 months), a significant VBG was achieved—from an initial 4.0 mm height and 3.7 mm width to 10.9 mm height and 5.3 mm width, thus allowing for successful implant placement [36].

The articles that follow the SLM method for membrane fabrication—specifically those by the teams of Geletu and Kurtis—present data from only a single patient, which is insufficient to draw definitive conclusions. However, the use of the SLM technique appears to result in a high success rate in bone augmentation.

In the study by Cucchi and colleagues, it is evident that the use of titanium membranes produced via the SLM method leads to successful outcomes. The study specifically compares two approaches: the use of the titanium mesh alone versus the mesh combined with a cross-linked collagen membrane.

The data indicated that, in terms of bone augmentation itself, there was no statistically significant difference between the two groups. However, regarding complications, the combined approach—mesh plus cross-linked collagen membrane—is preferred.

#### 4.1.3. Direct Metal Laser Sintering

The 2011 pilot clinical study by Ciocca et al. involved one patient with a residual atrophic maxillary arch. A customized titanium mesh was fabricated via DMLS, with a graft consisting of a mixture of autogenous and demineralized bovine bone. After a 6-month healing period, the mesh was removed, and the assessment showed a crestal bone gain of approximately 2.57 mm vertically and 3.41 mm horizontally, with no complications occurring [29].

Ciocca et al. also evaluated prosthetically guided bone augmentation of atrophic jaws using customized DMLS titanium meshes in 9 patients with partially or totally atrophic maxillae and mandibles. The membranes were loaded with a 1:1 mixture of autologous bone chips and organic bovine bone. After 6 to 8 months of healing, VBG ranged from 1.72 to 4.1 mm in the mandible and 2.14 to 6.88 mm in the maxilla. Mesh exposure occurred in 6 of 9 patients, with three early (within 4–6 weeks) and equally so delayed (10–24 weeks) exposures. In only one patient, the mesh was removed early due to pus exudation. Despite the high exposure rate, all planned implants (n = 26) were successfully placed according to the virtual plan, and no implant failures or complications were reported during prosthetic follow-up [30].

In the study of Cucchi et al., DMLS was also used to fabricate customized titanium meshes for vertical ridge augmentation. Ten participants in the clinical trial exhibited moderate to advanced vertical bone loss in the posterior maxilla or mandible. For grafting, autogenous bone and xenograft with peripheral venous blood were combined. Following recovery (6 to 8 months) and mesh removal, implants were inserted in the enhanced sites. The study showed an average vertical bone increase of 4.5 ± 1.8 mm and a mean regenerated bone volume of 892 mm^3^. The regeneration rate was 89% of the targeted bone volume. Surgical complications, mainly attributed to mesh exposure, occurred in three of ten cases, whereas healing complications were observed only in one patient [31].

According to all 10 of the studies, the average VBG is between 1.77 mm and 11.63 mm. Average HBG is between 1.6 mm and 10.34 mm. These parameters could be affected by complications, the type of bone graft, and the area of augmentation. Definite conclusions cannot be drawn because further clinical trials should be conducted.

### 4.2. Complication Rate

A study from 2011 gives a clear view of the complications and how they should be classified. According to the Fontana et al. classification, there are two main types of complications: surgical and healing. In each complication class, several subclasses can be distinguished as follows [39]:

The healing complications can be divided into four classes:

- Class 1—small membrane exposure (<3 mm) without purulent exudate;
- Class 2—large membrane exposure (>3 mm) without purulent exudate;
- Class 3—membrane exposure with purulent exudate;
- Class 4—abscess formation without membrane exposure.

Surgical complications are of three main types:

- Flap damage;
- Neurologic complication;
- Vascular complication.

The above-mentioned classification was used to provide more detailed information on the complications that appeared in three of the studies included in this review [33,37,38]. In four articles, the authors reported the complications without categorizing them [30,31,32,34], and the remaining three articles did not contain information on complications [29,35,36].

While most of the studies do not mention surgical complications [29,30,32,34,35,36,38], there are articles that pointed out the percentage of surgical complications and their type [31,33,37]. The most common among them was temporary paraesthesia (a neurological complication). Other mentioned complications during surgery were permanent paraesthesia and a hematoma [31,33,37]. One article also mentioned technical complications that occurred during the surgery (two partial mesh fractures and one partial mesh misfit) [33].

Healing complications were reported in every article relevant to this review. Three studies indicated that no complications occurred during the healing period [29,35,36]. In the other seven, the most common complication was stated to be mesh exposure, either early or delayed, with or without infection [30,31,32,33,34,37,38]. Moreover, five of these studies reported infection occurrence without mesh exposure [30,32,33,37,38]. Four articles described different treatment plans for managing these complications [30,33,34,37]. In the study of Cucchi et al., the sites with exposure were treated by mesh removal and local antibiotics. The flaps were closed without a membrane, and later, the implants were placed with additional GBR if needed [37]. In another study, Cucchi et al. examined the exposed meshes every 2 months and prescribed patients to use a soft brush and apply chlorhexidine digluconate 1% gel twice a day [30]. Dellavia et al. decided to maintain the meshes until the planned removal time in their study [34]. Finally, Cucchi et al. removed the early uncovered meshes one to three months post-surgery and extracted those with delayed exposure within seven days of the complication occurrence [33].

Taken together, these studies demonstrate that DMLS-manufactured titanium meshes offer a high degree of precision, mechanical stability, and predictability in bone regeneration procedures. However, mesh exposure remains a notable risk and appears to be a consistent complication across studies.

### 4.3. Type of Bone Graft Material

Although various types of graft materials were employed in the studies, a consistent element was the inclusion of autologous bone in 9 out of 10 articles [29,30,31,32,33,34,36,37,38]. Autografts are considered the gold standard for bone augmentation procedures due to their qualities. These grafts are biocompatible, osteoinductive, osteoconductive, and have osteogenic potential [40,41,42].

Despite being the paragon, autologous bone has some disadvantages. One of the major limitations of guided bone regeneration with autologous bone material is the limited bone volume available. Moreover, there are some concerns about postoperative morbidity. That is why autologous bone is not used in large bone defects [41,43].

In the present article, autologous bone is used in combination with xenograft in a 1:1 ratio. Xenografts can be prepared from many different sources, such as bovine, porcine, coral exoskeletons, and eggshells [40,44,45,46].

In all the included studies, except for that of Ciocca et al. [29], the exploited xenografts are of bovine origin. Xenografts provide a scaffold that supports new bone formation on the structure of the animal-derived graft [47]. They are mainly used because of their availability and osteoconductive potential [48].

One of the herein included articles reported the use of a combination graft consisting of allogenic and xenogeneic bone substitutes [35]. An allograft is a biological material obtained from a donor of the same species but a different individual. To ensure their safety for clinical use, allogeneic grafts undergo various types of processing and sterilization procedures [49]. They have excellent osteoinductive capabilities. Although they come from the same species, allografts have different genetic compositions, which can lead to discussions about immunological reactions and rejections, blood compatibility, and disease transmission [50,51].

In one study, the authors used a combination of autologous graft with alloplastic graft [29]. Alloplastic bone materials are synthetic materials that are used in dental implantology. Some of their advantages are biocompatibility, osteoconduction, easy manipulation, decreased risk of complications, immune reaction, and spreading of disease [52,53].

Some studies used platelet-rich plasma with a combination of graft materials [30,33,37].

The comparison between allograft and autograft use did not reveal a substantial difference in treatment success, indicating that the graft type may not be a critical factor in determining the effectiveness of the augmentation procedure.

When examining the amount of bone gain, we can conclude that the SLM method showed the greatest gain based on the reported average values (Table 5).

A limitation of the studies, however, is that not all of them provided data regarding HBG.

Also, we should mention that the sample size is another factor that cannot be ignored. The average results are based on only 32 patients treated with the SLM method compared to 96 treated with the SLS method.

Despite the limitations presented above, the most bone was gained with an augmentation procedure aided by individualized titanium meshes made with the SLM technique. It should be considered that two of the three articles using the SLM technique have only one patient, which should be considered a limitation. In the articles using meshes produced with SLS and DMLS techniques, there is no significant difference in the amount of bone volume gained.

The heterogeneity of the gained bone volume could have many different reasons. Different surgical techniques, the size of the bone defect, and patient specifics are factors that can impact the bone volume gained. Future studies should aim to analyze the impact and importance of each factor that can impact the results.

DMLS is a technique with fewer complications—surgical complications such as paresthesia, hematoma were observed, whereas in healing, exposure complications were reported, most of them being wound exposure.

In the SLM studies, two of the articles reported no complications, but in one study, complications arose in 16.67% of the cases during healing. Surgical complications were only observed in that particular investigation; the other two gave no information with regard to such problems, which is a limitation for the conclusions drawn. Compared with the previously mentioned results for DMLS, this could indicate that the SLM technique is actually less likely to lead to complications.

Given the data at hand, one could conclude that the SLS method would, however, show a higher rate of implied complications in comparison with DMLS. A variety of surgical and postoperative complications were reported with this method, the most frequent being mesh exposures. (Table 6).

However, we should keep in mind the number of patients presented in the studies, which can affect the complication rate. Most patients were treated with SLS-produced meshes (96 patients) compared to the number of patients treated with SLM (32 patients) and DMLS (20 patients). This can make a significant difference in the complication rate.

Although various types of graft materials were employed in the studies, a consistent element was the inclusion of autologous bone in all cases. In most of the clinical studies, a combination of autograft and xenograft was used (Table 7).

There were no significant differences in the type of bone graft used. The most common ratio used is a 1:1 ratio. One study that used the SLM method of production used allograft bone in combination with mineralized bovine bone.

In this systematic review, we discussed only studies using customized titanium mesh manufactured by SLM, SLS, and DMLS methods. The bone gain and possible complications were thoroughly analyzed. Previous systematic reviews on the topic considered the use of individualized and conventional titanium meshes [10,54]. To the best of our knowledge, this is the first systematic review that discussed the use only of customized titanium meshes made with SLM, SLS, and DMLS methods. The review aimed to describe, analyze, and summarize the current information on augmentation with individualized titanium meshes manufactured by SLM, SLS, or DMLS methods.

The major limitations of this systematic review were the small number of studies that met the inclusion criteria. In addition, the risk of bias assessment was not conducted on three studies because it is not applicable. Another limitation is the insufficient sample size in some of the studies.

Future directions should aim for more clinical trials to evaluate the success rate of the customized titanium meshes in guided bone regeneration. Factors that impact the gained bone volume should be analyzed in depth. While complications are commonly observed, further studies should aim to analyze how their percentage could be reduced.

## 5. Conclusions

Lack of bone is one of the most frequently encountered problems in modern dental implantology. The advancement in digital dental medicine, especially in additive manufacturing, makes planning and individualizing the process of guided bone regeneration more effective and accessible. This systematic review discusses and evaluates the results achieved with augmentation using customized titanium meshes manufactured with SLS, SLM, or DMLS methods. These augmentation methods demonstrated promising results in terms of gained bone volume. The results shown when comparing the bone volume gained gave a clear edge to the meshes produced with the SLM technique over the SLS and DMLS techniques. Despite this, it cannot be concluded that this method is superior due to the small sample size treated with SLM meshes. Also, the limited sample size in most of the studies should be considered as a limitation, which could affect the gained bone volume. Complications were commonly observed in most of the studies. The percentage of complications should be taken into account and further studied. Future research should examine the percentage of complications and how it can be reduced. In the results shown, most of the studies used autologous bone graft in combination with xenograft or alloplastic material. Autologous bone graft is the preferred material for this augmentation technique. The method of individualizing the meshes could be used for any case that has pre-operative preparation, achieving around 90% bone gain compared to planned bone volume and high stability of the implants. The available data was insufficient for this method of augmentation to be considered preferable over others. The results mentioned in this review are promising and should be further studied.

## Figures and Tables

**Table 1:** Results concerning bone gain and type of bone graft from the original articles included in the present study.

Authors	Sample Size	Vertical Bone Gain (VBG)	Horizontal Bone Gain (HBG)	Type of Bone Graft
Ciocca et al., 2011 [29]	1 patient	2.57 mm	3.41 mm	Autologous bone with alloplastic material 1:1 ratio
Ciocca et al., 2018 [30]	9 patients	1.72 mm to 4.1 mm in mandible, 2.14 mm to 6.88 mm in maxilla	n.i.	Autologous bone with an organic bovine bone 1:1
Cucchi et al., 2020 [31]	10 patients	4.5 ± 1.8 mm	n.i.	Autologous bone and xenograft with peripheral venous blood 1:1 ratio
Chiapasco et al., 2021 [32]	41 patients	4.78 mm	6.35 mm	Autologous bone with bovine bone material 1:1 ratio
Cucchi et al., 2021 [33]	30 patients	4.74 mm in the group where customized titanium mesh was solely applied, 6.36 mm in the group where the mesh was covered with collagen membrane	n.i.	Autologous bone and xenograft with peripheral venous blood 1:1 ratio, with or without collagen membrane
Dellavia et al., 2021 [34]	20 patients	5.20 mm	6.80 mm	Autologous bone with DBBM with collagen membrane
Geletu et al., 2022 [35]	1 patient	11.63 mm length	10.34 mm width	Allograft bone with pure mineral bovine bone 1:1 ratio
Kurtis et al., 2023 [36]	1 patient	6.9 mm	1.6 mm	Autologous bone with DBBM in a 50:50 ratio and i-PRF
Cucchi et al., 2024 [37]	48 patients	5.18 ± 1.61 mm	n.i.	Autologous bone and xenograft with peripheral venous blood 1:1 ratio
Giragosyan et al., 2024 [38]	20 patients	3.65 ± 1.73 mm	2.48 ± 1.03 mm	Autologous bone with xenograft 1:1 ratio

n.i.—no information.

**Table 2:** Results concerning complications (surgical and healing) from the original articles included in the present study.

Authors	Sample Size	Healing Time	Surgical Complications	Healing Complications
Ciocca et al., 2011 [29]	1 patient	8 months	No complication	No complication
Ciocca et al., 2018 [30]	9 patients	6 ÷ 8 months	n.i.	3 premature exposure, 3 delayed exposure, 1 removed with infection
Cucchi et al., 2020 [31]	10 patients	6 ÷ 8 months	30% (one permanent paresthesia, one temporary paresthesia, one hematoma)	10% (early exposure without infection)
Chiapasco et al., 2021 [32]	41 patients	5 ÷ 12 months (average 7.3 months)	n.i.	In 11 out of 53 sites (ca. 21%; 3 sites with limited exposure, 3 sites with exposure and minimal bone loss, 3 sites with partial bone loss, 1 site with early mesh removal, one site with failed restoration)
Cucchi et al., 2021 [33]	30 patients	6 months	4 neurological lesions (paresthesia), 3 technical complications (2 partial mesh fractures and 1 partial mesh misfitting) 13.3% the group where customized titanium mesh was solely applied; 26.7% in the group where the mesh was covered with collagen membrane	5 mesh exposures (3 early and 2 late exposures) and 2 infections without exposure Healing complications did not show statistical differences between the two study groups
Dellavia et al., 2021 [34]	20 patients	9 months	n.i.	3 mesh exposures (2 early exposures, 1 late exposure) without infection
Geletu et al., 2022 [35]	1 patient	6 months	No complications	No complications
Kurtis et al., 2023 [36]	1 patient	9 months	No complications	No complications
Cucchi et al., 2024 [37]	48 patients	6 months—mandible 9 months—maxilla	12% (one flap perforation and two temporary paresthesia)	8.3% (one case of point-like exposure with exudate, one case of late abscess without exposure)
Giragosyan et al., 2024 [38]	20 patients	At least 6 months	n.i.	33.3%

n.i.—no information.

**Table 3:** Risk of bias assessment for case reports.

JBI Checklist Questionnaire	Geletu et al., 2022 [35]	Ciocca et al., 2011 [29]	Kurtis et al., 2023 [36]
1. Were patient’s demographic characteristics clearly described?	Yes	Yes	Yes
2. Was the patient’s history clearly described and presented as a timeline?	Yes	Yes	Yes
3. Was the current clinical condition of the patient on presentation clearly described?	Yes	Yes	Yes
4. Were diagnostic tests or assessment methods and the results clearly described?	Yes	Yes	Yes
5. Was the intervention(s) or treatment procedure(s) clearly described?	Yes	Yes	Yes
6. Was the post-intervention clinical condition clearly described?	Yes	Yes	Yes
7. Were adverse events (harms) or unanticipated events identified and described?	Yes	Unclear	Yes
8. Does the case report provide takeaway lessons?	Yes	Yes	Yes

**Table 4:** Risk of bias assessment for case series.

JBI for Case Series	Dellavia et al., 2021 [34]
Were there clear criteria for inclusion in the case series?	Yes
Was the condition measured in a standard, reliable way for all participants included in the case series?	Yes
Were valid methods used for identification of the condition for all participants included in the case series?	Yes
Did the case series have consecutive inclusion of participants?	Unclear
Did the case series have complete inclusion of participants?	Yes
Was there clear reporting of the demographics of the participants in the study?	Yes
Was there clear reporting of clinical information of the participants?	Yes
Were the outcomes or follow-up results of cases clearly reported?	Yes
Was there clear reporting of the presenting site(s)/clinic(s) demographic information?	Yes
Was statistical analysis appropriate?	Yes

**Table 5:** Comparison of the bone gained.

Method	Reference	Bone Gain	Average
**DMLS**	Cucchi et al., 2020 [31]	4.5 ± 1.8 mm VBG	3.62 mm VBG 3.41 mm HBG
	Ciocca et al., 2011 [29]	2.57 mm VBG 3.41 mm HBG	
	Ciocca el al., 2018 [30]	1.72 mm to 4.1 mm in mandible 2.14 mm to 6.88 mm in maxilla	
**SLS**	Cucchi et al., 2024 [37]	5.18 ± 1.61 mm VBG	3.402 VBG 4.415 ± 1.03 HBG
	Chiapasco et al., 2021 [32]	4.78 mm VBG 6.35 mm HBG	
	Giragosyan et al., 2024 [38]	3.65 ± 1.73 mm VBG 2.48 ± 1.03 mm HBG	
	Dellavia et al., 2021 [34]	5.20 mm VBG 6.80 mm HBG	
**SLM**	Geletu et al., 2022 [35]	11.63 mm length 10.34 mm width	7.407 VBG 5.97 HBG
	Kurtis et al., 2023 [36]	6.9 mm VBG 1.6 mm HBG	
	Cucchi et al., 2021 [33]	4.74 mm VBG in group M− 6.36 mm VBG in group M+	

**Table 6:** Comparison of the complication rate.

Method	Reference	Complication Rate	Average
**DMLS**	Cucchi et al., 2020 [31]	10% due to early exposure without infection 30% surgical complication rate	25.3% exposure 10% complication rate 3.66% removed with purulent infection
	Ciocca et al., 2011 [29]	No complications	
	Ciocca et al., 2018 [30]	3 premature exposures 3 delayed exposures 1 removed with purulent infection	
**SLS**	Cucchi et al., 2024 [37]	8.3% healing complication 12% surgical complications	19.8% complication rate
	Chiapasco et al., 2021 [32]	Complications in 11 out of 53 sites occurred 15 to 150 days after the surgery	
	Giragosyan et al., 2024 [38]	33.3% complication rate	
	Dellavia et al., 2021 [34]	3 mesh exposures (2 early and 1 delayed exposure)	
**SLM**	Cucchi et al., 2021 [33]	3 early exposures 2 late exposures 2 infections without exposure 3 technical complications	11.111% complication rate
	Kurtis et al., 2023 [36]	No complications	
	Geletu et al., 2022 [35]	No complications	

**Table 7:** Comparison of the type of bone graft used.

Method	Reference	Type of Bone Graft	Average
**DMLS**	Cucchi et al., 2020 [31]	Autologous bone and xenograft with peripheral venous blood 1:1 ratio	100% autologous bone in combination with a secondary grafting material in a 1:1 ratio
	Ciocca et al., 2011 [29]	Autologous bone with alloplastic material 1:1 ratio	
	Ciocca et al., 2018 [30]	Autologous bone with an organic bovine bone 1:1	
**SLS**	Cucchi et al., 2024 [37]	Autologous bone and xenograft with peripheral venous blood 1:1 ratio	100% autologous bone in combination with a xenograft in a 1:1 ratio
	Chiapasco et al., 2021 [32]	Autologous bone with bovine bone material 1:1 ratio	
	Giragosyan et al., 2024 [38]	Autologous bone with xenograft 1:1 ratio	
	Dellavia et al., 2021 [34]	Autologous bone with DBBM with collagen membrane	
**SLM**	Cucchi et al., 2021 [33]	Autologous bone and xenograft with peripheral venous blood 1:1 ratio, with or without collagen membrane	66.66% autologus bone with 1:1 xenograft 33.33% allograft with xenograft
	Kurtis et al., 2023 [36]	Autologous bone with DBBM in a 50:50 ratio and i-PRF	
	Geletu et al., 2022 [35]	Allograft bone with pure mineral bovine bone 1:1 ratio	

## Data Availability

The original contributions presented in this study are included in the article/Supplementary Materials. Further inquiries can be directed to the corresponding author.

## Supplementary Materials

The following supporting information can be downloaded at https://www.mdpi.com/article/10.3390/dj13090387/s1. Figure S1: Traffic light chart for risk of bias assessment for RCT studies; Table S1: Prisma checklist.

## Abbreviations

The following abbreviations are used in this manuscript:

CAD	Computer-aided design
CAM	Computer-aided manufacturing
AM	Additive manufacturing
PBF	Powder bed fusion
LPBF	Laser powder bed fusion
EBM	Electron beam melting
DED	Direct energy deposition
SLS	Selective laser sintering
SLM	Selective laser melting
DMLS	Direct metal laser sintering
OSF	Open science framework
