# Supplementary figures and images for: Influence of SLM-, SLS-, and DMLS-Manufactured Titanium Meshes on Bone Gain Parameters and Complications: A Systematic Review
Source: Dent J (Basel). 2025 Aug 26;13(9):387. doi: 10.3390/dj13090387 (PMC12468853; doi:10.3390/dj13090387)

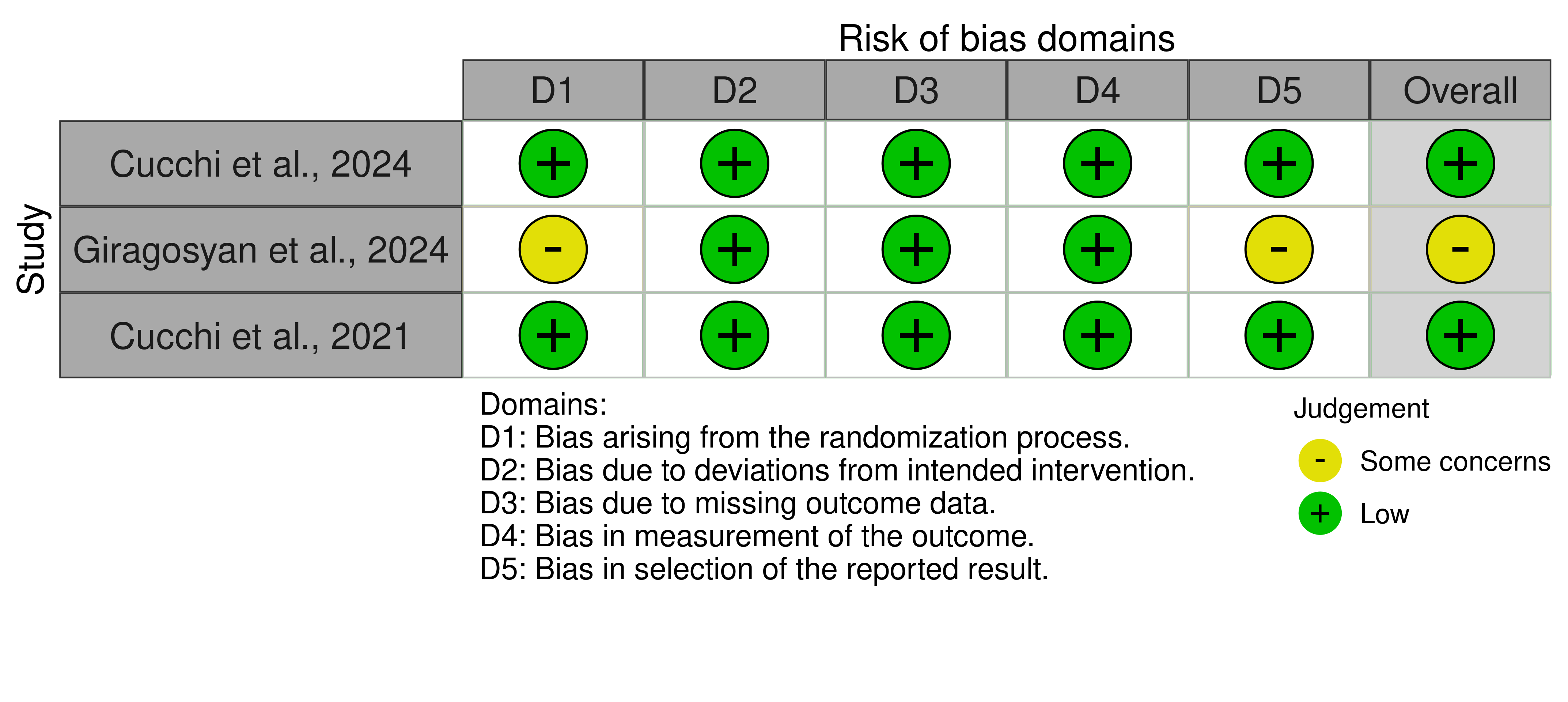

Supplement: Supplementary file 1 [file dentistry-13-00387-s001.zip › Figure S1.png]
